# Photochemical and Patternable Synthesis of 2D Covalent Organic Framework Thin Film Using Dynamic Liquid/Solid Interface

**DOI:** 10.1002/smtd.202400063

**Published:** 2024-05-09

**Authors:** Taewoong Kim, Joohee Oh, Seung Cheol Kim, Jong‐Guk Ahn, Soyoung Kim, Young Yong Kim, Hyunseob Lim

**Affiliations:** ^1^ Department of Chemistry Gwangju Institute of Science and Technology (GIST) Gwangju 61005 Republic of Korea; ^2^ Analysis and Assessment Group Research Institute of Industrial Science and Technology Pohang 37673 Republic of Korea; ^3^ Beamline Division, Pohang Accelerator Laboratory Pohang University of Science & Technology Pohang 37673 Republic of Korea

**Keywords:** 2D COFs, flow cell, liquid/solid interface, patternable, ultra‐smooth film

## Abstract

2D covalent organic frameworks (COFs) are highly porous crystalline materials with promising applications in organic electronics. Current methods involve either on‐surface synthesis (solid surface) or interfacial synthesis (liquid/liquid, liquid/gas interface) to create thin films for these applications, each with its drawbacks. On‐surface synthesis can lead to contamination from COF powder or unreacted chemicals, while interfacial synthesis risks damaging the film during post‐transfer processes. These challenges necessitate the development of alternative synthesis methods for high‐quality 2D COF films. This study presents a novel approach for synthesizing homogeneous 2D COF thin films by combining photochemistry and a liquid‐flowing system. Leveraging previous work on liquid flow systems to prevent contamination during solvothermal synthesis, this approach to the photochemical method, resulting in the synthesis of high‐crystalline 2D COF films with tunable thickness is adopted. The photochemical approach offers spatially controllable energy sources, enabling patternable COF synthesis. Notably, it is successfully fabricated ultrasmooth patterned 2D COF films on hexagonal boron nitride, offering a streamlined process for optoelectronic device fabrication without additional pre, post‐processing steps.

## Graphical abstract

This study presents a novel method for creating high‐quality 2D covalent organic framework (COF) films. Combining photochemistry and a liquid‐flowing system mitigates contamination and film damage concerns, yielding homogeneous, high‐crystalline 2D COF films with adjustable thickness. This streamlined approach enables patternable COF synthesis, demonstrated by fabricating ultrasmooth 2D COF films on hexagonal boron nitride for simplified optoelectronic device production.

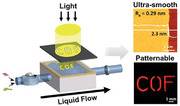

## 1 Introduction

Covalent organic frameworks (COFs) are crystalline porous materials formed by the robust covalent bonding of light weight elements.^[^ ^1^ ^]^ Types of COFs are classified as 2D and 3D COFs according to the crystal structures. Notably, organic units in 2D COFs are covalently linked into 2D atomic layers, and they stacked into layered crystal structure via π–π interaction. Owing to their distinctive attributes such as low weight density, high permanent porosity and extensive surface area coupled with exceptional thermal and chemical stability,^[^ ^2^, ^3^, ^4^ ^]^ they have risen as the appropriate candidates beyond the traditional amorphous organic polymers for the wide range of applications like organic field effect transistors,^[^ ^5^, ^6^ ^]^ optoelectronic devices,^[^ ^7^, ^8^, ^9^, ^10^ ^]^ and ion‐selective membranes.^[^ ^11^, ^12^ ^]^ However, powder form of COF synthesized by conventional methods is limited for its practical applications due to low solubility and limited processability. To address these issues, new synthetic strategies aimed at producing large area 2D film (or membrane) forms of 2D COF have been explored.^[^ ^13^, ^14^, ^15^, ^16^, ^17^, ^18^, ^19^, ^20^ ^]^ The developed methods typically employ strategies to selectively enhance the reactivity between organic building blocks at surface or interface, such as solid surfaces (referred to as *on‐surface synthesis*) or at liquid/liquid and liquid/gas interfaces (referred to as *interfacial synthesis*). Although these techniques have enabled the substantially uniform 2D COF films, several issues should be further addressed; 1) contaminating adsorption of 2D COF powder or unreacted chemical species formed in liquid phase during *on‐surface synthesis*, and 2) physical damage to the film during post transfer process during *interfacial synthesis*.

We herein demonstrate the innovative approach that combines photochemical processes with a liquid‐flow system to synthesize ultrasmooth 2D COF thin films. Our previous studies demonstrated the photochemical route for synthesizing 2D COF power or films for the first time.^[^ ^21^ ^]^ In photochemical reactions, the light source not only supplies adequate energy to surpass the activation barrier but also facilitates reaction pathways that are not accessible through thermally driven reactions. An additional advantage of photochemical synthesis especially for solid phase products is the spatially resolved reaction controllability, which facilitates the patterned synthesis only on the targeted region where the light source is irradiated. Liquid flow systems have been used to synthesize COFs using the solvothermal method to prevent contamination by accumulation of unreacted monomers or polymers on the film.^[^ ^22^ ^]^ By integrating these two approaches, i.e., i) photochemical reaction and ii) liquid flow system,^[^ ^16^, ^21^ ^]^ we have effectively synthesized the ultrasmooth and highly crystalline 2D COF films with controlled thickness on sapphire or hexagonal boron nitride (h‐BN). The growth of 2D COF can be spatially controlled simply by utilizing a photomask, which facilitates the patterned growth that was previously unachievable with liquid/liquid or liquid/gas interfaces. These traditional methods were hindered by diffusion and the unstable floating nature of the COF film. This breakthrough in COF film synthesis paves the way for the direct fabrication of optoelectronic devices, offering a promising avenue for high‐quality, patterned 2D COF films suitable for a range of technological applications.

## 2 Results and Discussion

### 2.1 Synthesis of 2D COF Films using Different Methods

**Scheme 1:**
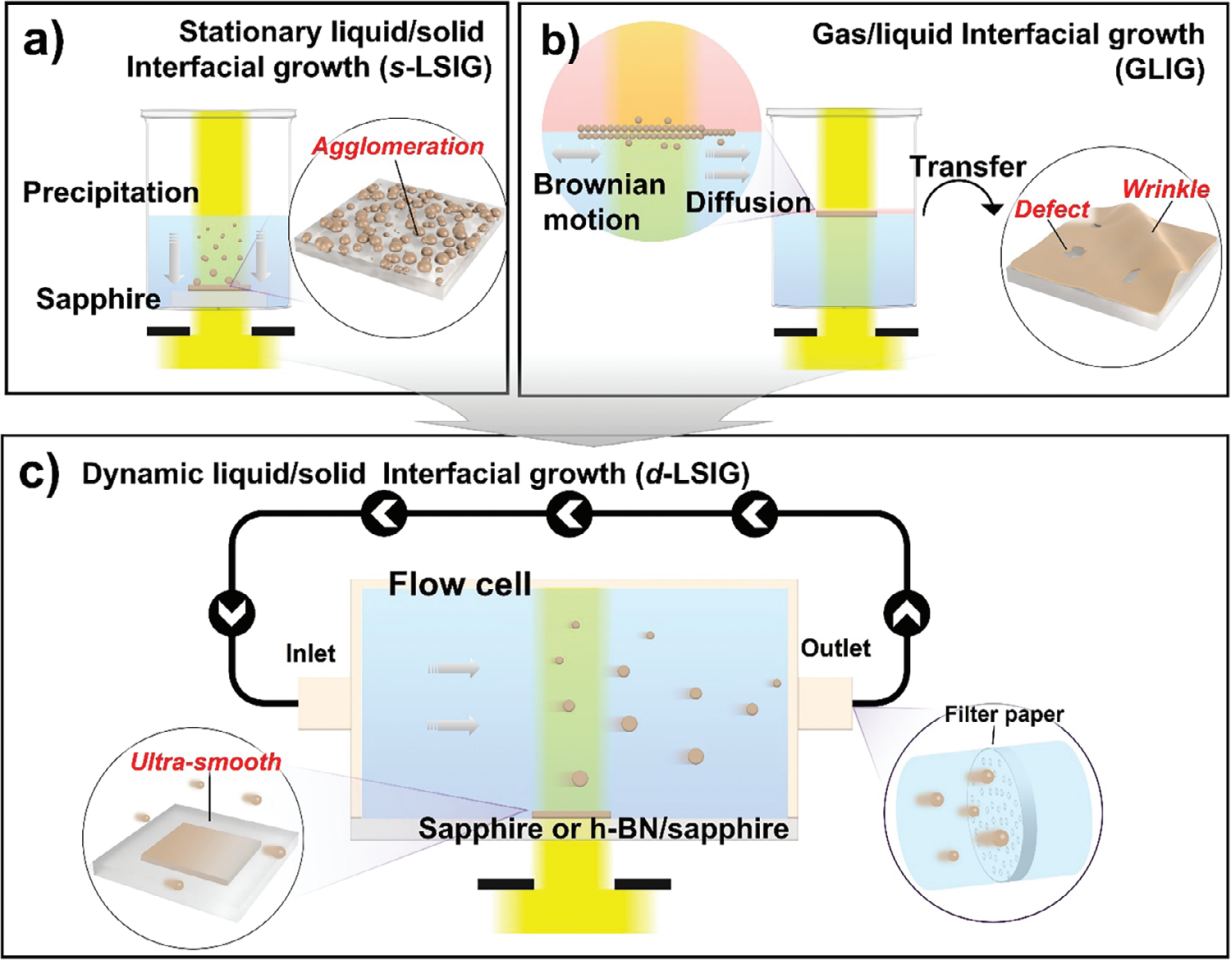
Schematic comparison of c) dynamic liquid/solid interfacial growth (*d*‐LSIG) method developed in this work with previous representative methods: a) stationary liquid/solid interfacial growth (*s*‐LSIG) and b) gas/liquid interfacial growth (GLIG).

**Figure 1:**
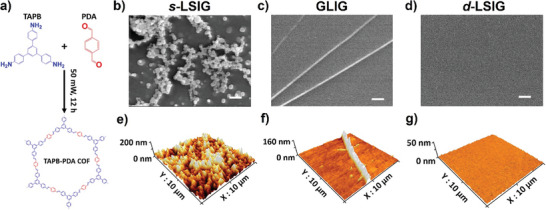
a) Molecular structure of TAPB‐PDA COF obtained by imine condensation reaction between TAPB and PDA. SEM images of COF films by b) *s*‐LSIG (scale bar = 200 nm), c) GLIG (scale bar = 10 µm) and d) *d*‐LSIG (scale bar = 200 nm). AFM images of COF films by e) *s*‐LSIG, f) GLIG and g) *d*‐LSIG.

To reassess the constraints associated with the current approaches to synthesizing 2D COF film, we undertook the synthesis of 2D COFs at the stationary interfaces, either between a liquid and a solid (**Scheme** **1**a) or between liquid and air (Scheme 1b), employing unit molecules such as 1,3,5‐tris(4‐aminophenyl) benzene (TAPB) and terephthalaldehyde (PDA) (**Figure** **1**a). The first attempted approach involves utilizing a solid surface within a static liquid solution without any dynamic flow, which is referred to as stationary liquid/solid interfacial growth abbreviated as *s*‐LSIG. The procedure is briefly described in Scheme 1a.^[^ ^1^, ^21^ ^]^ The second attempted method used an air/liquid interface by floating all precursors on the liquid surface, referred to as air (gas)/liquid interfacial growth denoted as GLIG (Scheme 1b).^[^ ^16^, ^17^, ^18^ ^]^ Scanning electron microscope (SEM) images in Figure 1b,c show the surface morphology of TAPB‐PDA COF synthesized by *s*‐LSIG and GLIG methods, respectively. On the TAPB‐PDA COF sample by *s*‐LSIG, particle‐like structures are observed (Figure 1b). In contrast, the smooth surface is confirmed on the COF sample prepared by GLIG method, although the wrinkled structures and cracked regions are observed (Figure 1c). The undesired particles on 2D COF by *s*‐LSIG might be originated by the reaction in the bulk solution which cannot be inhibited in this approach. Despite GLIG method can suppress the reaction in the bulk solution, an inevitable post‐transfer process onto the target solid substrate is required, which causes the undesired wrinkled structures in COF films. Another limitation of GLIG method is that the patternable synthesis is not available, because the Brownian motion of liquid surface and diffusion of initially formed oligomers and polymers to the unexposed area cannot be prohibited on the liquid surface.

**Figure 2:**
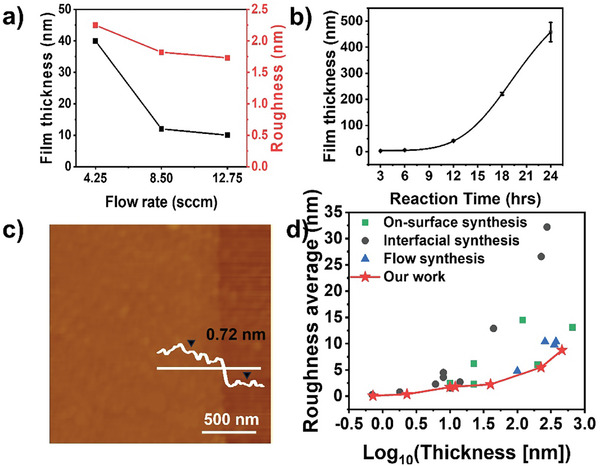
a) Variation in film thickness and roughness average of the COF film with different flow rate. b) Film thickness distribution of the COF films over various reaction times. c) AFM image of a monolayer COF film. d) Comparative analysis with previous studies.

To address two challenges posed by the aforementioned approaches: 1) inhibiting the undesired growth of particles in the liquid solution, and 2) necessity of the rigid interfacial template, we developed the innovative method that utilize the dynamic liquid/solid interface exploiting the flow cell system as described in Scheme 1c, which is referred to as dynamic liquid/solid interfacial growth (denoted as *d*‐LSIG) (See Supporting Information for detailed experimental procedures). While the rigid liquid/solid interface can be used with this approach for photochemical synthesis of 2D COF, the small particles initially forming in the bulk liquid phase can be successfully removed by flowing liquid and filter system (Figure S1, Supporting Information). The temperature of solution was maintained at the room temperature. It is noteworthy that the elevation in the temperature causes the formation of COF particles even in the precursor solution not only on the surface, which prohibits the liquid flow by clogging the filter. Consequently, significantly clean surface without any undesired structure is observed on the TAPB‐PDA COF film synthesized by *d*‐LSIG as confirmed in SEM image in Figure 1d. 3D atomic force microscope (AFM) images (Figure 1e–g) more clearly show the difference in the surface topology. Compared to the other two methods (R_a_ = 37.75 (*s*‐LSIG) nm and 8.17 (GLIG) nm) (Figure 1e,f), ultrasmooth surface topology (R_a_ = 2.25 nm) is confirmed on the TAPB‐PDA COF film by *d*‐LSIG (Figure 1g). Another noteworthy benefit of photochemical synthesis of 2D COF film at dynamic liquid/solid interface lies in its tunability regarding thickness of 2D COF film. To demonstrate this, we have investigated the effect of reaction time, and flow rate on the film quality and thickness, respectively. First, the flow rate was regulated with all other parameters such as precursor concentrations, reaction time and light intensity held constant. The thickness of 2D COF films depending on the flow rate was examined using AFM. With an increase in flow rate, TAPB‐PDA COF film exhibited a gradual reduction in thickness (**Figure** **2**a; Figure S1, Supporting Information), accompanied by a decrease in surface roughness, indicating improved film quality (Figure 2a). We suggested that increasing the flow rate would result in the higher pressure exerted on the unreacted chemical species that were bonded to the film surface, which caused an increased separating efficiency from the film surface leading to better film quality. Meanwhile, precursor residence time at the surface of target substrate would decrease at the same time, which may reduce the number of molecules staying for enough time to react by interacting with the substrate or existing COF film. With an increase in the reaction time, the thickness of the 2D COF film exhibited an exponential increase, because precursor's interaction with the substrate to form the film and its interaction with the film for growth are different (Figure 2b; Figure S2, Supporting Information). Following these findings, we typically employed a flow rate exceeding 12.75 sccm, while regulating the reaction time to fine‐tune the thickness of the 2D COF film. In practice, the film thickness can be manipulated within a range spanning from a few nanometers to several tens of nanometers. Impressively, we managed to synthesize an ultra‐ thin TAPB‐PDA 2D COF film, achieving a thickness of just a single monolayer under meticulously controlled conditions (Figure 2c). We conducted a comparative analysis of the film thickness and surface roughness values with those of 2D COF films reported in previous literatures (Figure 2d).^[^ ^18^, ^22^, ^23^, ^24^, ^25^, ^26^, ^27^, ^28^, ^29^, ^30^ ^]^

We also explored synthesizing films using different types of COFs and substrates to demonstrate the versatility of our method, which utilizes a dynamic liquid/solid interface for creating high‐quality films. Two other 2D COF films, COF‐5 (boron‐linked COF) and Tp‐TAPB COF (amine‐linked COF), were successfully synthesized by our approach. 2,3,6,7,10,11‐hexahydroxytriphenylene and 1,4‐benzene diboronic acid were used as precursors for COF‐5, and 1,3,5‐triformylphloroglucinol (Tp) and TAPB for Tp‐TAPB were used for Tp‐TAPB COF (Figures S3a and S4a, Supporting Information). Characterizations by infrared (IR) spectroscopy and AFM confirmed their high quality of all COF films synthesized by our method, although the thickness of the films varied depending on the types of precursors (Figures S3b,c and S4b,c, Supporting Information). To demonstrate the versatility across different substrate types, TAPB‐PDA COF films were synthesized on both quartz and glass substrates (Figure S5, Supporting Information). Although the smooth and high quality COF films were synthesized on both substrates, it was observed that films on these substrates were thicker compared to those on sapphire substrates under identical conditions. This thickness variation is likely attributable to differences in substrate absorbance and the interactions between the monomer and substrate during the synthesis process. Nonetheless, the surface roughness of these films, in comparison to their thickness, remained low, similar to those synthesized on the sapphire substrate (Figure 2d). These results indicate that employing a dynamic liquid/solid interface can improve film quality, regardless of the COF type or substrate utilized.

### 2.2 Characterizations of TAPB‐PDA 2D COF films

**Figure 3:**
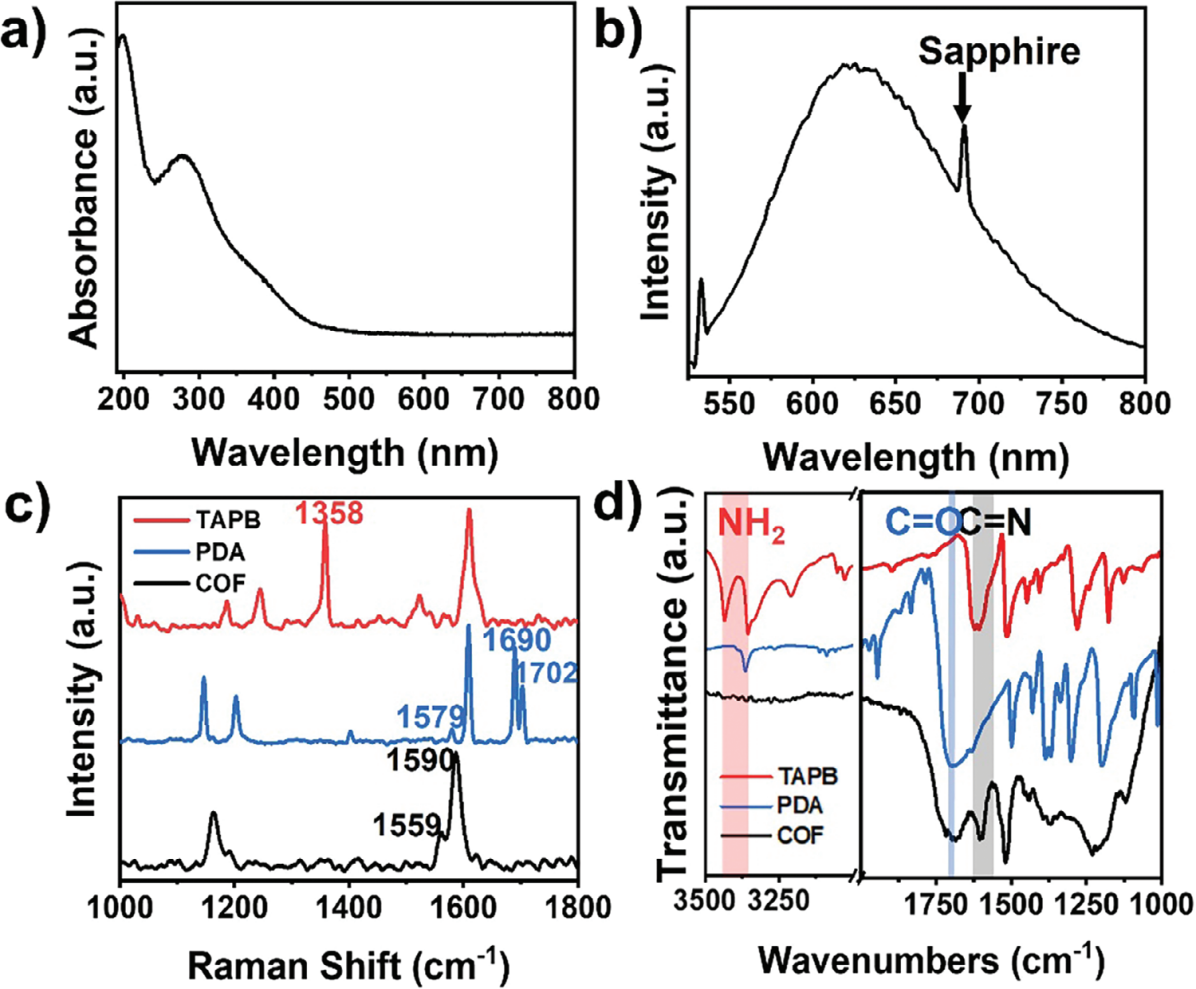
Spectroscopic characterization of crystalline TAPB‐PDA COF film. a) UV–vis spectrum of the COF film on the C‐plane sapphire. b) PL spectrum of the COF film on the C‐plane sapphire. c) Raman spectra and d) IR spectra of precursor molecules, TAPB (red), PDA (blue) and the produced COF film (black).

Spectroscopic analyses were then carried out to characterize the chemical structure of TAPB‐PDA COF film by *d‐*LSIG process. In the UV–vis absorption spectrum (**Figure** **3**a), a strong absorption peak appears at 277 nm, which corresponds to aromatic π to π* transition (Figure S3, Supporting Information). Photoluminescence (PL) spectrum was then obtained by using the excitation with a 532 nm laser (Figure 3b), in which a strong emission peak was observed at ≈625 nm, corresponding to HOMO‐LUMO energy gap. These optical characteristics of TAPB‐PDA 2D COF film are also consistent with the results in previous literature.^[^ ^18^ ^]^ The formation of imine (R_1_‐N = R_2_) bonds by the condensation reaction between primary amine (‐NH_2_) and aldehyde groups (‐CHO) of organic units was confirmed both by Raman and IR spectroscopies. In the Raman spectra of TAPB (red line in Figure 3c) and PDA (blue line in Figure 3c), we observed characteristic vibrational modes: the β‐ring vibration mode (1358 cm^−1^) and the NH_2_ wagging mode (1579 cm^−1^) for TAPB molecules, as well as the benzene ring stretching mode (1690 cm^−1^) and the C = O stretching mode (1702 cm^−1^) for PDA molecules. These Raman peaks of unit molecules disappeared in the Raman spectrum of TAPB‐PDA 2D COF film (black line in Figure 3c), while strong peaks newly appeared at 1559 cm^−1^ and 1590 cm^−1^, corresponding to the vibration modes of the aromatic ring chain and the C = N stretching mode. The IR spectra also provide evidence of the imine formation in TAPB‐PDA COF by *d*‐LSIG (Figure 3d). The amine stretching bands of TAPB (3434 and 3355 cm^−1^) and the C = O stretching band (1690 cm^−1^), which were evident in the IR spectra of individual molecules (represented by red and blue lines in Figure 3d), were absent in the IR spectrum of the TAPB‐PDA 2D COF (depicted in black in Figure 3d). Conversely, new peaks corresponding to imine stretching modes (1621, 1596, and 1563 cm^−1^) were detected. The disappearance of precursor‐related peaks and the appearance of COF‐related peaks in Raman and IR spectra affirm the successful synthesis of TAPB‐PDA 2D COF through the photochemical reaction between TAPB and PDA precursors.

### 2.3 Characterizations of TAPB‐PDA 2D COF Films on h‐BN

**Figure 4:**
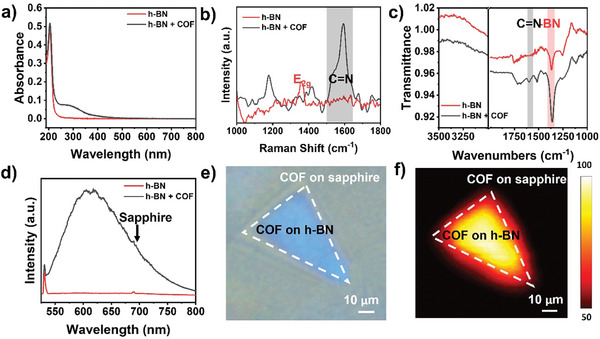
a) UV–vis spectrum of h‐BN (red) and the COF film on the h‐BN (black). b) Raman spectra of h‐BN (red) and the COF film on the h‐BN (black). c) IR spectra of h‐BN (red) and the COF film on the h‐BN (black). d) PL spectrum of h‐BN (red) and the COF film on the h‐BN (black). e) Optic image of TAPB‐PDA 2D COF on the h‐BN sheet. f) PL image of TAPB‐PDA 2D COF on the h‐BN sheet.

**Figure 5:**
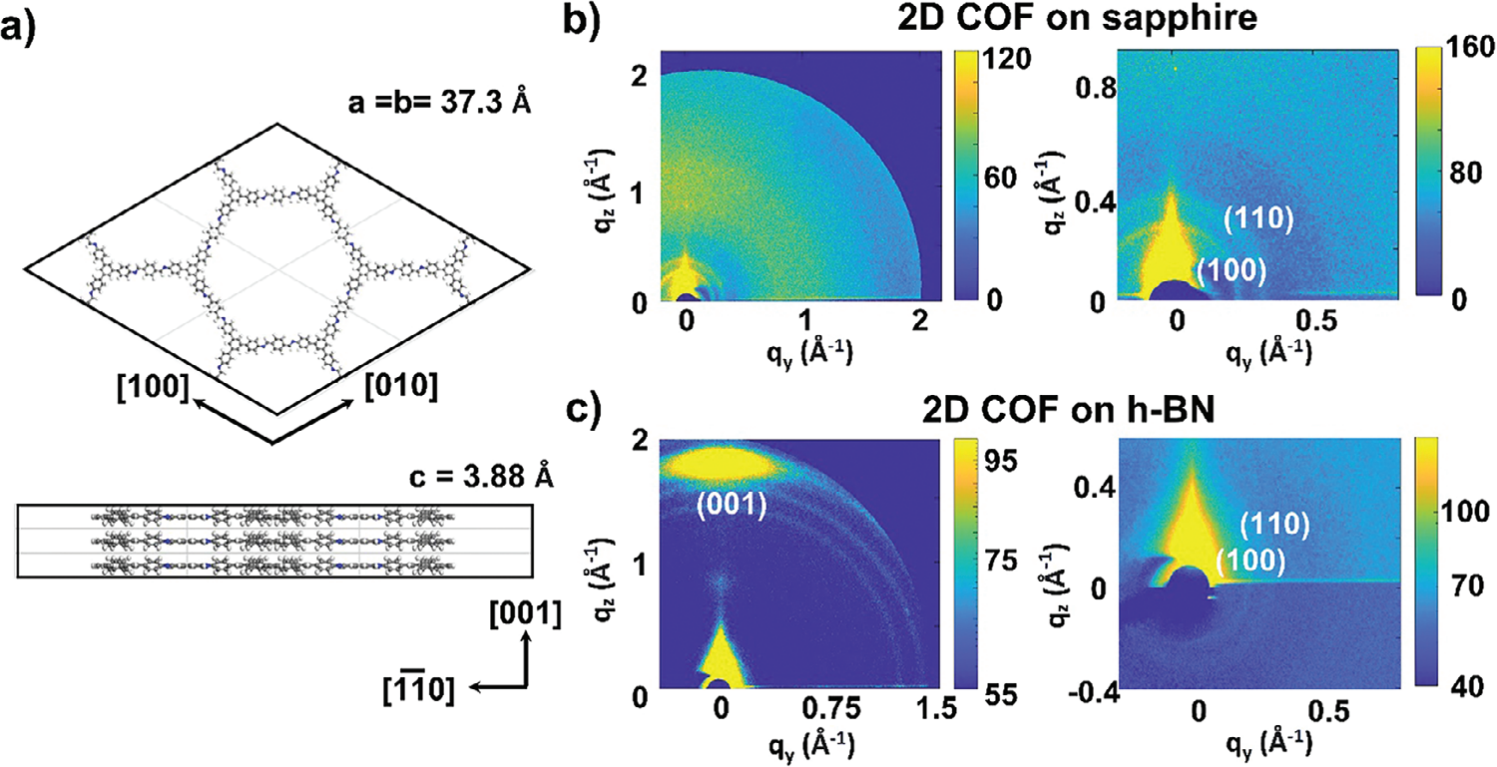
a) Eclipsed stacking crystal structure of TAPB‐PDA COF. b) GI‐WAXS images of TAPB‐PDA COF film on the C‐plane sapphire substrate (left) and the magnified image (right). c) GI‐WAXS images of TAPB‐PDA COF film on the h‐BN (left) and the magnified image (right).

Recently, 2D nanomaterials such as graphene and hexagonal boron nitrides (h‐BN) have been employed as the substrates for 2D COF film synthesis to promote selective crystal orientation.^[^ ^6^, ^31^ ^]^ In this study, we also attempted to use h‐BN sheets as target solid substrates (**Figure** **4**a) for improving crystallographic orientation selectivity. It is noteworthy that we chose the h‐BN as a 2D material substrate instead of graphene, since 2D COF film grown on insulating h‐BN substrate can be directly used for electrical device applications. Spectroscopy characteristics of TAPB‐PDA 2D COF film grown on chemical vapor deposition (CVD) grown h‐BN substrates ^[^ ^32^ ^]^ by *d*‐LSIG are shown in Figure 4a–d. Absorption, Raman, IR, and PL characteristics of COF on h‐BN closely resemble those of the COF grown on a c‐plane sapphire substrate (Figure 3). However, PL intensity of COF on h‐BN is notably higher than that of COF on sapphire. The normalized PL intensity, relative to the sapphire phonon mode, was enhanced by more than 6 times when utilizing h‐BN substrate (Figure 4d). In general, defect states in electronic structure caused by disordered crystal structure provide non‐radiative recombination paths for exciton, leading to the reduction of PL. The significant enhancement in PL was further investigated by synthesizing COF on a c‐plane sapphire substrate, where small h‐BN flakes by mechanical exfoliation were placed, as depicted in Figure 4e. PL mapping on this sample demonstrates the robust PL measured on COF grown on a h‐BN flake in comparison to that measured on COF grown on the surrounding sapphire region (Figure 4f). We believe that the enhanced PL intensity would be attributed to the improved crystallinity induced by outstanding orientation selectivity on the h‐BN substrate. To study the crystallographic characteristics of TAPB‐PDA 2D COF film grown on sapphire or h‐BN substrate by *d*‐LSIG method, grazing incident wide angle X‐ray scattering (GI‐WAXS) was performed using synchrotron X‐ray source with an incident angle of 0.21°. Previous literatures including our work have suggested the eclipsed stacking crystal structure of TAPB‐PDA COF rather than the staggered stacking crystal structure as shown in **Figure** **5**a. GI‐WAXS pattern obtained from TAPB‐PDA 2D COF (80 nm thick) on sapphire (Figure 5b) shows the two distinct ring patterns at q_z_ = 0.21 and 0.37 Å^−1^, which can be assigned to (100) and (110) planes of TAPB‐PDA COF crystal. The obtained GI‐WAXS pattern is well matched with the simulated spot positions of GI‐WAXS (q_xy_ (100) = 0.195, q_xy_ (110) = 0.337 Å^−1^) (Figure S6, Supporting Information). The crystal structure observed in the high‐resolution transmission electron microscope (TEM) images of TAPB‐PDA 2D COF is also consistent with the GI‐WAXS results (Figure S7, Supporting Information). The presence of diffraction patterns corresponding to the (100) and (110) planes provide evidence for the development of a crystalline framework structure in the TAPB‐PDA 2D COF film through the *d*‐LSIG process. However, the occurrence of a uniform ring‐shaped diffraction pattern simultaneously indicates that, at least in thicker COF films, polycrystalline traits are prominent, with no discernible preference for a specific crystallographic orientation on the sapphire substrate. However, the GI‐WAXS pattern obtained from TAPB‐PDA 2D COF film on h‐BN sheet (Figure 5c) shows a notably intense spot at q_z_ = 1.80 Å^−1^ corresponding to the interlayer d‐spacing of 3.49 nm, indicating (001) plane diffraction was observed only in the surface normal direction. This distinguished result implies that the face‐on orientation of TAPB‐PDA 2D COF is highly preferred on the h‐BN sheets owing to the π–π stacking interaction between TAPB‐PDA 2D COF and h‐BN. These crystallographic results demonstrated that 2D h‐BN is an excellent substrate for the synthesis of highly crystalline 2D COF films.

### 2.4 Patterned Synthesis of TAPB‐PDA 2D COF Films Using *d*‐LSIG Method

**Figure 6:**
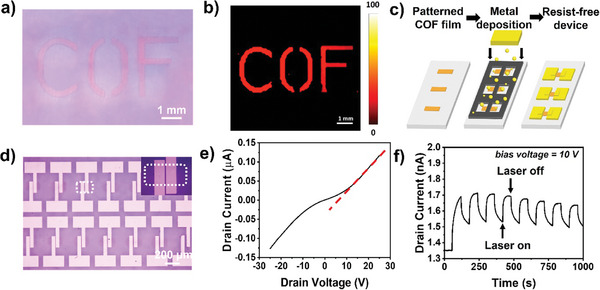
a) Optical image and b) PL mapping image of capital‐‘COF’‐patterned TAPB‐PDA 2D COF film. c) Fabrication scheme of resist‐free device. d) Optical image of electrical device array (dotted line indicated the patterned COF region). e) IV characteristic curve of the TAPB‐PDA COF film. f) Photocurrent response of TAPB‐PDA COF film.

In pursuit of our ultimate objective in this work, the photochemical and patternable synthesis of a 2D COF film, we developed the patterned synthesis system employing an optical mask. **Figure** **6**a,b shows the optical image and PL mapping image of patterned TAPB‐PDA 2D COF film with the pattern of capital letters, “COF”. These results suggest that our approach has the strong advantage in the patternable synthetic manner. The capability of patterned synthesis of 2D COF film in this work also enables the “resist‐free” device fabrication as shown in Figure 6c. TAPB‐PDA 2D COF film was first synthesized exclusively within the channel regions on h‐BN/sapphire substrate by patternable *d*‐LSIG process, then metal electrodes were deposited through the “shadow mask” by conventional thermal evaporation method. Overall, an electrical device array could be fabricated only with a two‐step procedure without the time‐ and cost‐consuming lithographic process (Figure 6d). The electrical and optoelectrical properties of TAPB‐PDA 2D COF film were then studied (Figure S4, Supporting Information). Electrical conductance was measured as 5.33 ± 0.44 ×10^−9^ S. when the channel length was 6 µm, and it exhibited the photo response in electrical conductance (an increase of 16.5%) upon exposure to light of 405 nm laser (Figure 6e,f). These demonstrations suggest the potential of our approach, the patternable *d*‐LSIG method, in advancing optical applications based on 2D COF film.

## 3 Conclusion

In summary, our study presents an advanced method for synthesizing 2D COF films through spatially controllable photochemical reactions utilizing a dynamic liquid/solid interface. These resulting large‐area films exhibited exceptional surface quality and adjustable thickness, achieved through precise control of flow rates and reaction times. Crystallographic analysis revealed a preferred face‐on orientation of COF films on h‐BN, indicating enhanced crystallinity and potential advantages for optical and electrical properties. Furthermore, we showcased the capability of patterned synthesis using optical masks, streamlining the device fabrication process without the need for complicated lithographic steps. Photocurrent measurements suggest the potential of these devices in optical applications. We believe that our innovative approach provides a promising route for the precise and scalable production of high‐quality, tunable 2D COF films. This advancement paves the way for their widespread application across various fields.

## 4 Experimental Section

### Synthesis of 2D COF Films Using s, d‐LSIG Methods

52.5 mg of TAPB and 30 mg of PDA were added to a dioxane/mesitylene solution (4:1 v/v) containing 64 ml of dioxane and 16 ml of mesitylene. After being sonicated for 10 min, the solution was filtrated using a syringe filter with a pore size of 0.02 µm to eliminate any remaining undissolved reactants. Subsequently, 8 ml of distilled water and 12 ml of acetic acid were added, and the solution was used to synthesize COF films using *s, d*‐LSIG method. In the case of the *s*‐LSIG method, a substrate was put into the reaction solution and patterned light was incident through a shadow mask.

In the case of the *d*‐LSIG method, the solution vessel was connected to the flow cell that was covered with a substrate on the bottom side using a hose. The flow was allowed on one side of the substrate through the flow cell, and the film was synthesized through a photochemical method in which light was incident through the substrate with high transparency such as a sapphire substrate. The solution was circulated along the hose using a pump while stirring the precursor solution. After the precursor solution was circulated several times, 50 mW of light was irradiated from the xenon lamp in the perpendicular direction of the flow. In this process, a filter was used to prevent the circulation of bulk COFs synthesized in the precursor solution, allowing only the precursors dissolved in the solution to circulate.

### Synthesis of 2D COF Films Using GLIG Method

GLIG method was implemented according to the previously reported literature.^[^ ^18^ ^]^ 7 mg of TAPB and 4 mg of PDA were introduced into a 20 mL glass vial, followed by the addition of a total of 8 mL of a solution containing 1,4‐dioxane, mesitylene, and chloroform in a 1:1:2 volume ratio without argon purging. After sonication for 10 min, the solution underwent filtration using a syringe filter with a 0.02 µm pore size to eliminate any undissolved reactants. Subsequently, 0.6 mL of acetic acid was incorporated. The prepared precursor solution was meticulously deposited onto the surface of distilled water within a reaction glass vessel. Finally, light exposure for 1 h using a solar simulator was performed from the top of the reaction vessel. Upon completion of the reaction, the resulting 2D COF film was carefully transferred on a target substrate by lifting up the substrate holder.

### Synthesis of h‐BN on Sapphire Using CVD

The h‐BN was synthesized as previously reported literature.^[^ ^32^ ^]^ A sapphire substrate (c‐plane) was positioned at the center of a 2‐inch alumina tube chemical vapor deposition (CVD, Sciencetech) system, while ammonia borane (97% purity, Sigma–Aldrich) was situated in a sub‐chamber. The furnace was raised to 1400 °C with a flow of Ar gas (10 sccm) and H_2_ gas (10 sccm). Simultaneously, the sub‐chamber was heated to 130 °C to facilitate the evaporation of ammonia borane. The commencement of multi‐layer h‐BN growth on the sapphire substrate was triggered by opening a valve in the sub‐chamber. Following the valve opening, the source was supplied for a duration of 30 min. Throughout the growth process, the pressure was maintained at 0.1 Torr. Upon completion of the growth, the furnace was gradually cooled to room temperature under a mixture of Ar and H_2_ gases.

### Resist‐Free Device Fabrication and Electrical Properties Measurement

After synthesizing the patterned COF film on a sapphire substrate using a shadow mask, the chromium and gold metal were deposited at target thicknesses of 5 and 20 nm, respectively by thermal evaporation using a shadow mask that had an electrode pattern. The channel length of each electrode pattern ranges from 6 to 30 µm. Electrical property measurements were performed at 0.25 V intervals from −25 to 25 V in each patterned electrode. Photocurrent measurement was performed while turning on/off the laser at 1 min intervals with the bias voltage set to 10 V.

## Conflict of Interest

The authors declare no conflict of interests.

## Supporting information

Supporting Information

## Data Availability

The data that support the findings of this study are available from the corresponding author upon reasonable request.
