# Supplementary material for: Photochemical and Patternable Synthesis of 2D Covalent Organic Framework Thin Film Using Dynamic Liquid/Solid Interface
Source: Small Methods. 2024 May 9;8(12):2400063. doi: 10.1002/smtd.202400063 (PMC11672181; doi:10.1002/smtd.202400063)
Supplement: Supplementary file 1 — Supporting Information [file SMTD-8-2400063-s001.docx]

Supporting Information

Photochemical and Patternable Synthesis of 2D Covalent Organic Framework Thin Film Using Dynamic Liquid/Solid Interface

Taewoong Kim^1^, Joohee Oh^1^, Seung Cheol Kim^1^, Jong-Guk Ahn^1^, Soyoung Kim^2^, Young Yong Kim^3^ and Hyunseob Lim^1,^*

^1^Department of Chemistry, Gwangju Institute of Science and Technology (GIST), Gwangju 61005, Republic of Korea

^2^Analysis and Assessment Group, Research Institute of Industrial Science and Technology, Pohang 37673, Republic of Korea

^3^Beamline Division, Pohang Accelerator Laboratory, Pohang University of Science & Technology, Pohang 37673, Republic of Korea

E-mail: hslim17@gist.ac.kr

**Experimental Section**

**Materials and Instrumentation.**

All chemicals and reagents were purchased commercially and used without additional purification. 1,4­benzene diboronic acid (BDBA) and 1,3,5-triformylphloroglucinol (Tp) were obtained from Sigma Aldrich. 1,3,5-tris (4-aminophenyl) benzene (TAPB) and terephthalaldehyde (PDA) were obtained from Alfa Aesar. 1,4-dioxane was purchased from DAEJUNG CHEMICALS & METALS CO., Ltd (Siheung, Republic of Korea). Mesitylene and 2,3,6,7,10,11-hexahydroxytriphenylene (HHTP) were obtained from Tokyo Chemical Industry Co., Ltd. and acetic acid was obtained from Alfa Aesar. Deionized water was produced by a water purification system (HUMAN SCIENCE).

Synthesis of COF film was carried out by irradiating light from Sciencetech Arc Lamp System LH series Lamp Housing & SF Solar Simulator. Especially, dynamic-liquid/solid interfacial growth (*d*-LSIG) method was conducted through BT100-2J Peristaltic Pump Drive.

**Synthesis of 2D COF films using *d*-LSIG methods.**

In the case of COF-5, 48.4 mg of HHTP and 37 mg of BDBA were added to a dioxane/mesitylene solution (1:1 v/v) containing 50 ml of dioxane and 50 ml of mesitylene. After being sonicated for 10 minutes, the solution was filtrated using a syringe filter with a pore size of 0.02 µm to eliminate any remaining undissolved reactants and the solution was used to synthesize COF films using *d*-LSIG method.

In the case of Tp-TAPB COF, 31.4 mg of Tp and 52.5 mg of TAPB were added to a dioxane/mesitylene solution (4:1 v/v) containing 64 ml of dioxane and 16 ml of mesitylene. After being sonicated for 10 minutes, the solution was filtrated using a syringe filter with a pore size of 0.02 µm to eliminate any remaining undissolved reactants. Subsequently, 8 ml of distilled water and 12 ml of acetic acid were added, and the solution was used to synthesize COF films using *d*-LSIG method.

**Characterizations.**

The field emission scanning electron microscope (SEM) images were obtained using a JSM-7500F microscope. The atomic force micrographs (AFM) images, thickness, and roughness of samples were acquired using a XE-100 equipment. The UV-vis spectra were acquired using an Agilent 8453 spectrometer. The Raman and PL (mapping) measurements were performed with a lab-made 532 nm green laser. The IR spectra were obtained at ATR mode using a Vertex 70V spectrometer. Grazing incidence wide-angle X-ray scattering (GI-WAXS) was performed at the Beamline 3C in the Pohang Accelerator Laboratory. The electrical characteristics of the resist-free device was measured with a Keithy 2400 analyzer under normal pressure conditions.

**Computational details**

Density functional theory computations were conducted using the Perdew-Burke-Ernzerhof (PBE)functional^[1]^ and Grimme’s D3 method to account for dispersion corrections.^[2]^ These methods were integrated into the Vienna Ab-initio Simulation Package(VASP).^[3, 4]^ The core electrons were treated by projector-augmented wave pseudopotentials,^[5]^ expanded within a plane wave basis set up to an energy cutoff of 500 eV. Ionic relaxations were executed until atomic forces reached values below 0.01 eV/Å (10^-7^ eV). A 1 × 1 × 10 Γ-centered grid was used for k-point sampling in Brillouin zone.

**Simulation Method for Grazing incidence X-ray diffraction pattern**

The indexing of grazing incidence X-ray diffraction was calculated using a, b, c, α, β, γ parameters and following formula:^[6, 7]^

The GIXS (*I*_GIXS_) can be expressed by the following formula:

$I_{\text{GIXS}}(\alpha_{f},2\theta_{f})\cong\frac{1}{16\pi^{2}}\frac{1-\exp[-2Im (q_{z})t]}{2Im (q_{z})}\left[ \begin{matrix} \left| T_{i}T_{f} \right|^{2}I_{1}(q_{xy},Re (q_{1z}))+ \\ \left| T_{i}R_{f} \right|^{2}I_{1}(q_{xy},Re (q_{2z}))+ \\ \left| R_{i}T_{f} \right|^{2}I_{1}(q_{xy},Re (q_{3z}))+ \\ \left| R_{i}R_{f} \right|^{2}I_{1}(q_{xy},Re (q_{4z})) \end{matrix} \right]$ (1)

where Re(*x*) and Im(*x*) represent the real and imaginary part of a complex number *x*, *t* is the film thickness, *R*_i_ and *T*_i_ are the reflected and transmitted amplitudes of the incoming X-ray beam, respectively, and *R*_f_ and *T*_f_ are the reflected and transmitted amplitudes of the outgoing X-ray beam, respectively. In addition, $q_{xy}$ is given by $q_{xy}=\sqrt{q_{x}^{2}+q_{y}^{2}}$. Further,$q_{1z}=k_{z,f}-k_{z,i}$, $q_{2z}=-k_{z,f}-k_{z,i}$, $q_{3z}=k_{z,f}+k_{z,i}$, and$q_{4z}=-k_{z,f}+k_{z,i}$; here, *k_z_*_,i_ is the *z*-component of the wave vector of incident X-ray beam, given by

$k_{z,i}=\frac{2\pi}{\lambda}\sqrt{n_{R}^{2}-\cos^{2}\alpha_{i}}$ (2)

and *k_z_*_,f_ is the z-component of the wave vector of outgoing X-ray beam, given by

$k_{z,f}=\frac{2\pi}{\lambda}\sqrt{n_{R}^{2}-\cos^{2}\alpha_{f}}$ (3)

where *λ* is the wavelength of the incident X-ray beam, *n_R_* is the refractive index of the film given by $n_{R}=1-\delta+i\xi$ with dispersion coefficient δ and absorption coefficient ξ. *α_i_* is the out-of-plane grazing incident angle of the incoming X-ray beam, and *α*_f_ is the out-of-plane exit angle of the outgoing X-ray beam. Here, *q_x_*, *q_y_*, and *q_z_* are the components of the scattering vector **q** and *I*_1_ is the scattering intensity of the structures in the film, which can be calculated kinematically. Moreover, for a thin film with a given orientation, its fundamental vectors can be rotated and transformed by a rotation matrix. When a structure in a thin film is randomly oriented in the plane of the film but uniaxially oriented out of plane, the peak position vector **q_c_** of a certain reciprocal lattice point **c*** in the sample reciprocal lattice is given by

$\boldsymbol{q}_{\boldsymbol{c}}\equiv(q_{c,x}, q_{c,y}, q_{c,z})=\boldsymbol{R}\cdot\boldsymbol{c}^{*}$ (4)

where **R** is a 3×3 rotation matrix to decide the preferred orientation of the structure in the film, and *q_c_*_,_*_x_*, *q_c_*_,_*_y_*, and *q_c_*_,_*_z_* are the *x*, *y*, and *z* components of the peak position vector **q_c_**, respectively. Using the above equation, every peak position can be obtained. Because of cylindrical symmetry, the Debye-Scherrer ring composed of the in-plane randomly oriented **c*** cuts the Ewald sphere at two positions in its top hemisphere: $q_{c,xy}\left( \equiv\pm\sqrt{q_{c,x}^{2}+q_{c,y}^{2}} \right)$ and $q_{c,z}$. Thus, diffraction patterns with cylindrical symmetry are easily calculated in the *q*-space. It is then convenient to determine the preferred orientation of known structures and further to analyze anisotropic X-ray scattering patterns. However, since *q*-space is distorted in GIXS by refraction and reflection effects, the relation between the detector plane expressed as the Cartesian coordinate defined by two perpendicular axes (*i.e.*, by the in-plane exit angle 2*θ*_f_ and the out-of-plane exit angle *α*_f_) and the reciprocal lattice points is needed. The two wave vectors *k_z_*_,i_ and *k_z_*_,f_ are corrected for refraction by the equations (2) and (3), respectively. Therefore, the two sets of diffractions that result from the incoming and outgoing X-ray beams are given at the exit angles by the following expression:

$\alpha_{f}=\cos^{-1} \sqrt{n_{R}^{2}-\left( \frac{q_{c,z}}{k_{0}}\pm\sqrt{n_{R}^{2}-\cos^{2}\alpha_{i}} \right)^{2}}$ (5)

Where $q_{c,z}/k_{0}>\sqrt{n_{R}^{2}-\cos^{2}\alpha_{i}}$. In equation (5), the positive sign denotes diffractions produced by the outgoing X-ray beam, and the negative sign denotes diffractions produced by the incoming X-ray beam. The in-plane incidence angle 2*θ*_i_ is usually zero, so the in-plane exit angle 2*θ*_f_ can be expressed as follows:

${2\theta}_{f}=\cos^{-1} \left[ \frac{\cos^{2}\alpha_{i}+\cos^{2}\alpha_{i}-\left( q_{c,xy}/k_{0} \right)^{2}}{2\cos\alpha_{i}\cos\alpha_{f}} \right]$ (6)


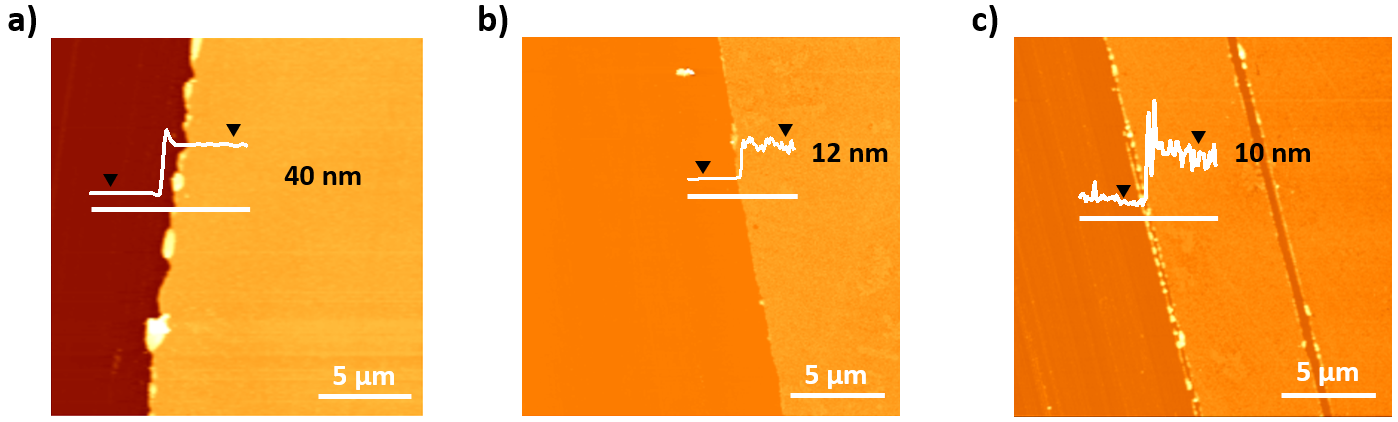


**Figure S1.** AFM images of the COF film at different flow rates. (a) 4.25 sccm, (b) 8.50 sccm, and (c) 12.75 sccm.


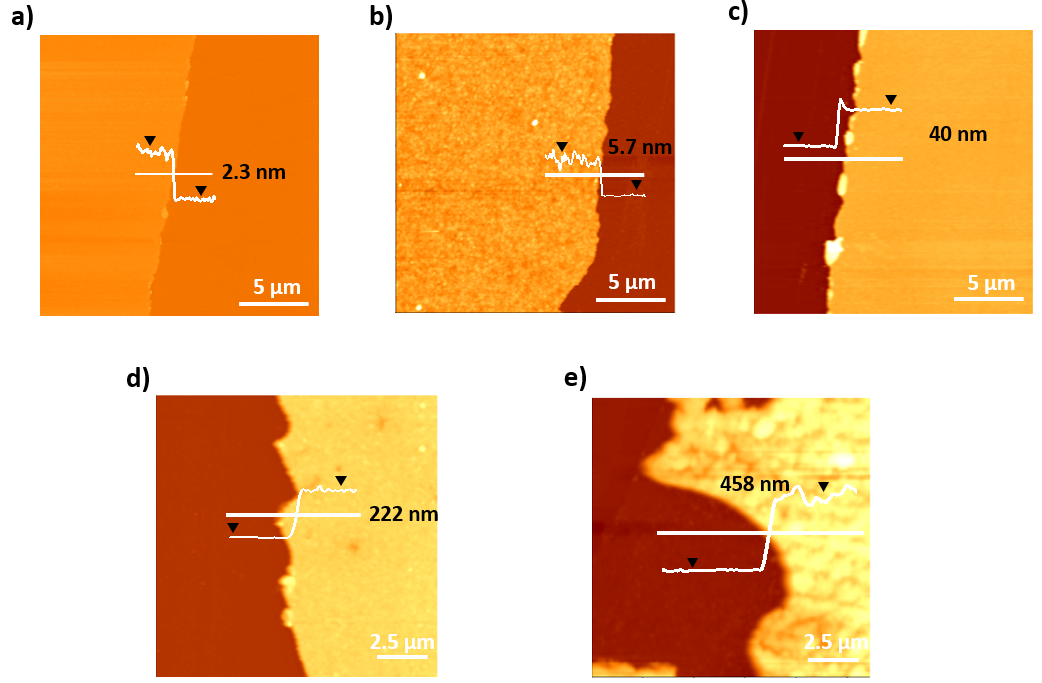


**Figure S2.** AFM images of the COF film at different reaction times. (a) 3 hours, (b) 6 hours, (c) 12 hours, (d) 18 hours, and (e) 24 hours.


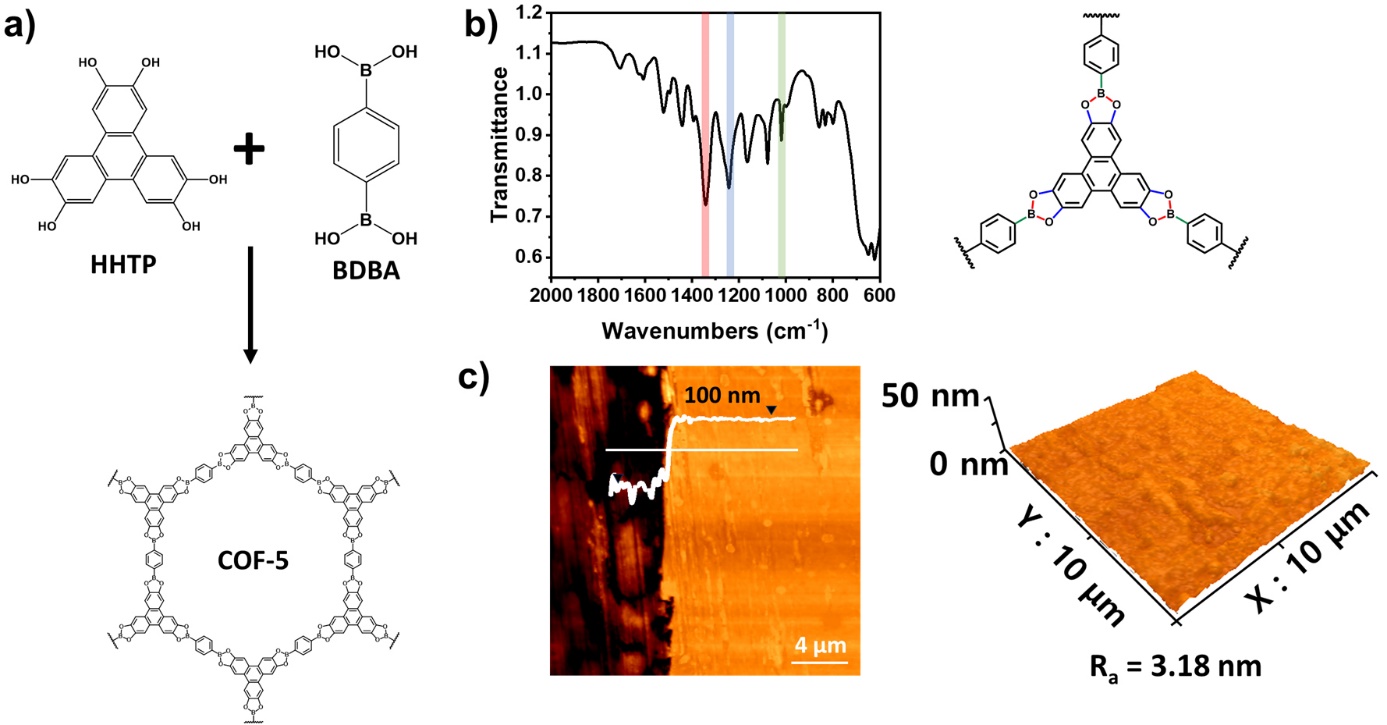


Figure S3. (a) Molecular structure of COF-5 obtained between HHTP and BDBA. (b) IR spectrum of COF-5 film. (c) AFM images of COF-5 film


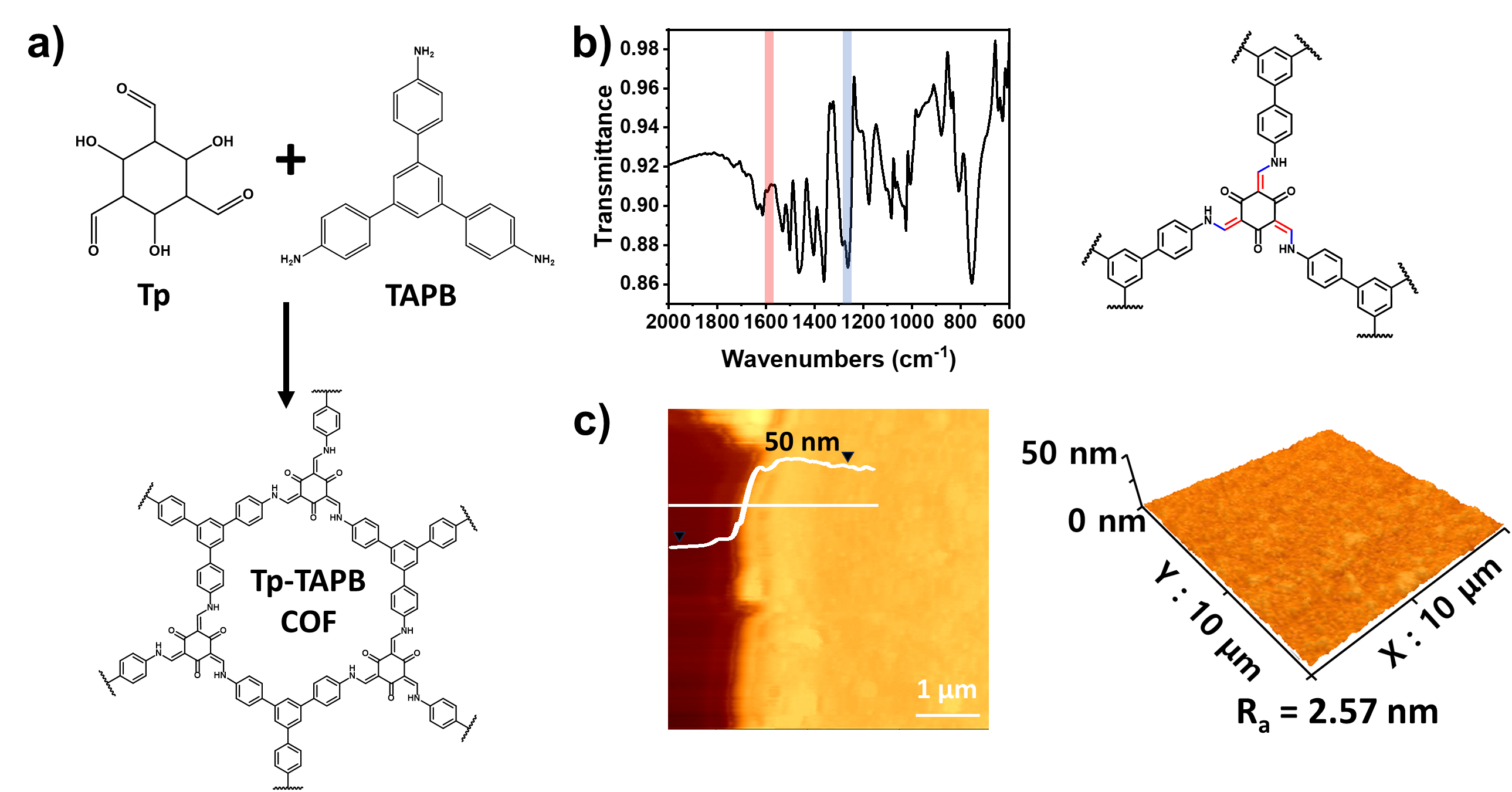


Figure S4. (a) Molecular structure of Tp-TAPB COF obtained between Tp and TAPB. (b) IR spectrum of Tp-TAPB COF film. (c) AFM images of Tp-TAPB COF film


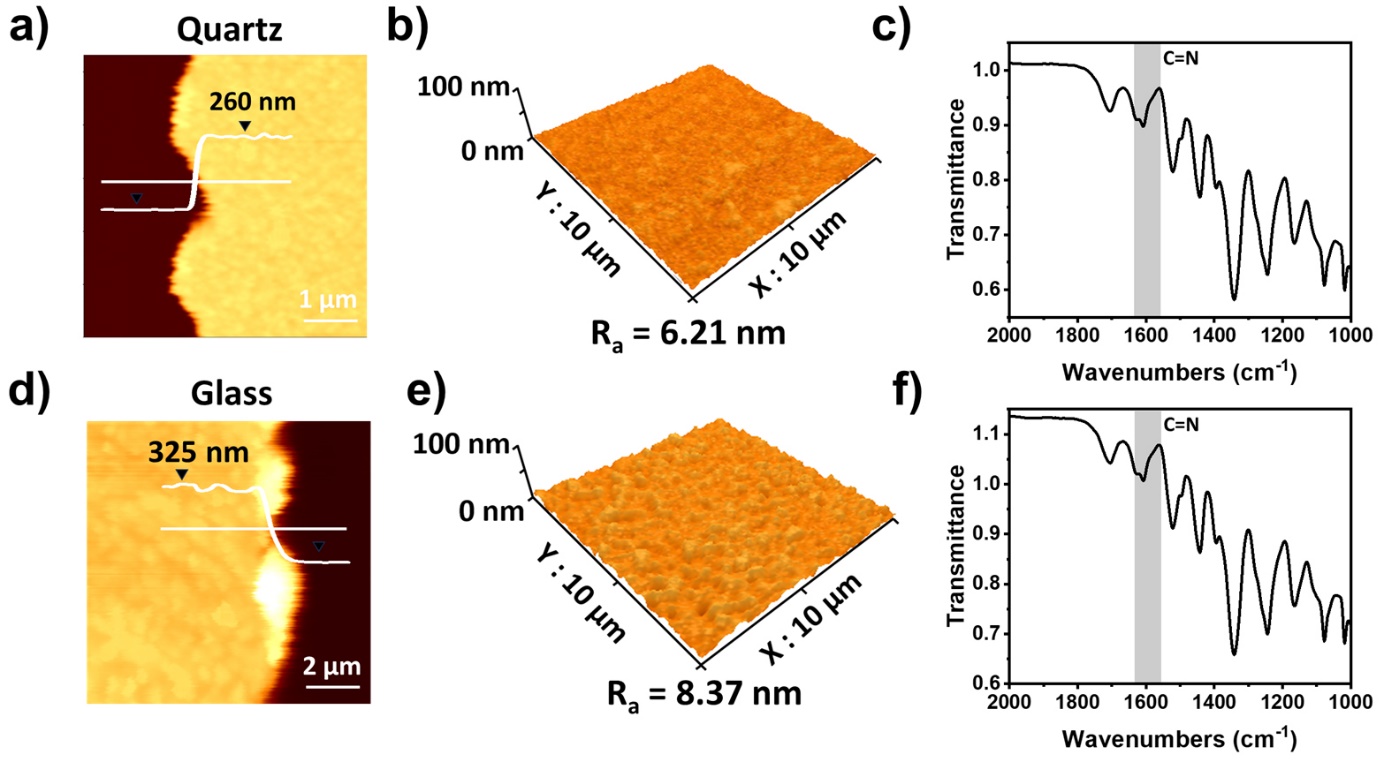


Figure S5. (a), (b) AFM images of the TAPB-PDA COF film synthesized on quartz substrate. (c) IR spectrum of the TAPB-PDA COF film synthesized on quartz substrate. (d), (e) AFM images of the TAPB-PDA COF film synthesized on glass substrate. (f) IR spectrum of the TAPB-PDA COF film synthesized on glass substrate.


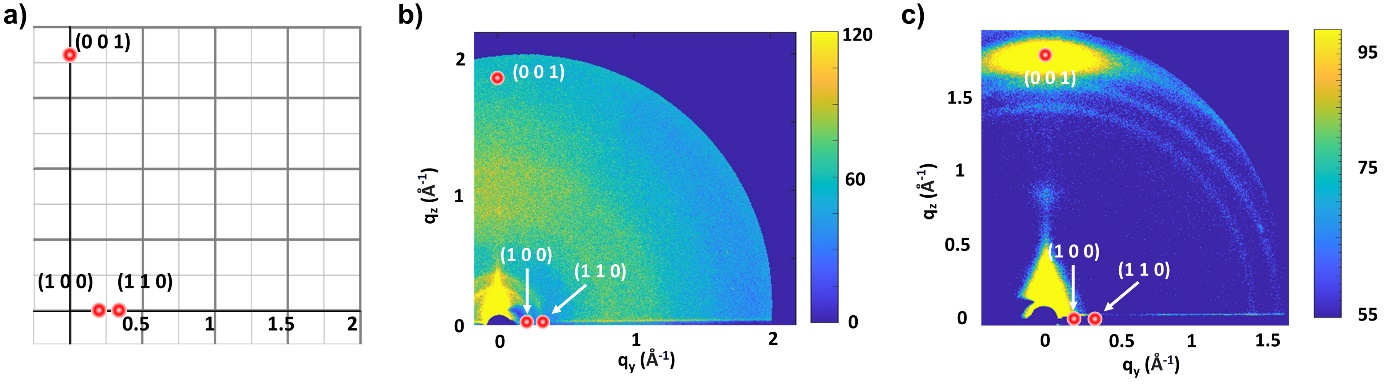


**Figure S6.** (a) Simulated GI-WAXS pattern. (b) Simulated GI-WAXS spot position on experimental GI-WAXS pattern of TAPB-PDA COF film on the C-plane sapphire substrate. (c) Simulated GI-WAXS spot position on experimental GI-WAXS pattern of TAPB-PDA COF film on the h-BN.


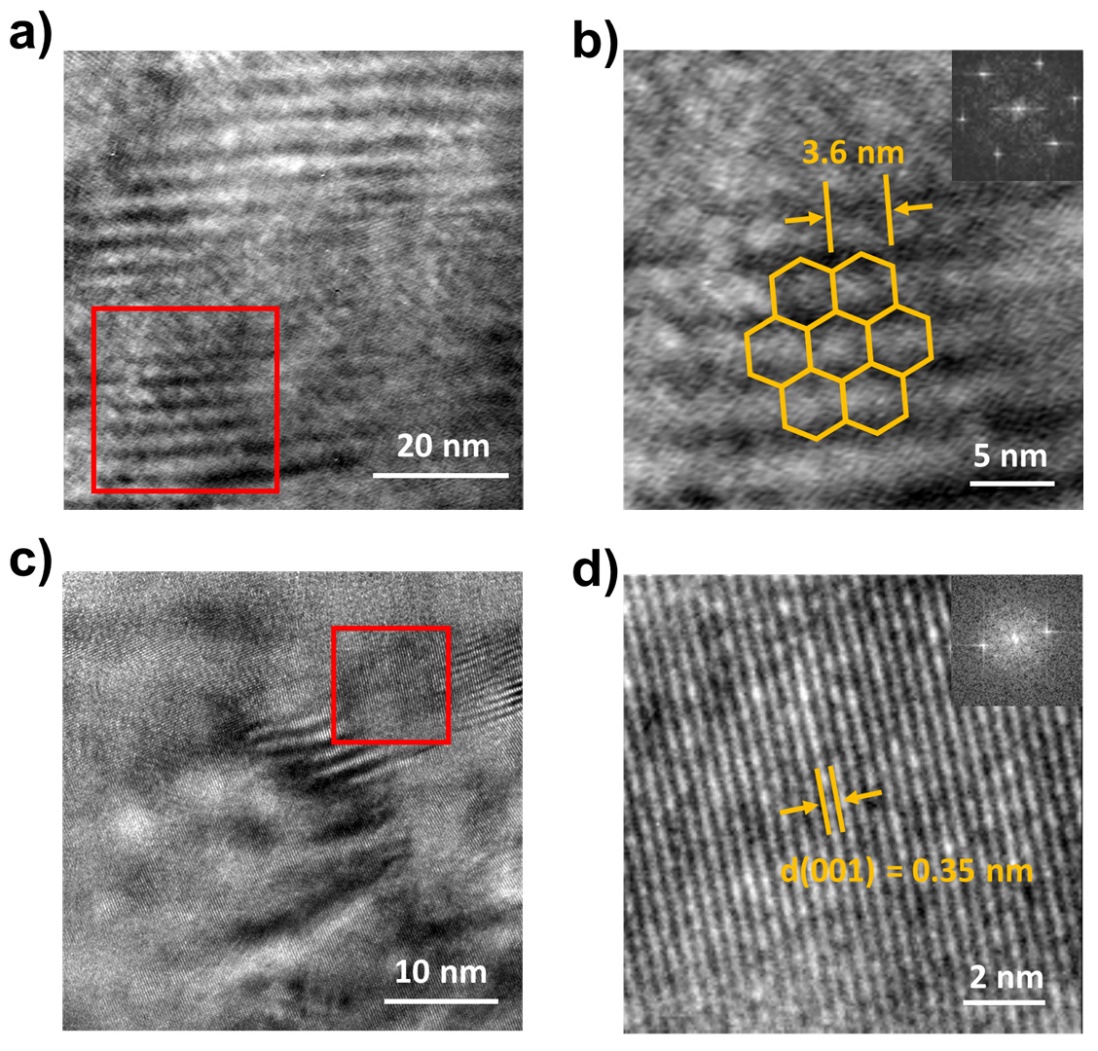


Figure S7. (a) High-resolution TEM image of hexagonal pores; (b) TEM image of TAPB-PDA COF giving a magnified view of the selected area. Inset: fast Fourier transform (FFT) from the image. (c) High-resolution TEM image of (001) area; (f) TEM image of TAPB-PDA COF giving a magnified view of the selected area. Inset: FFT from the image.


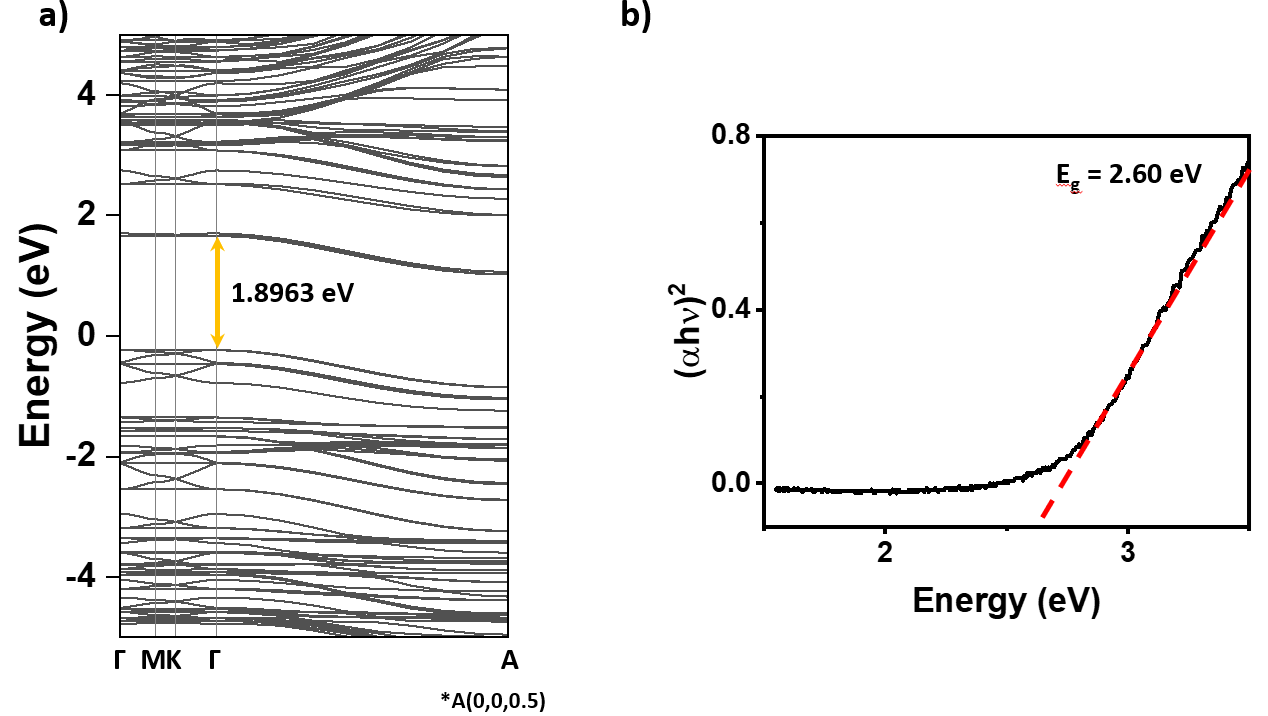


**Figure S8.** (a) Calculated energy band diagram. (b) Tauc plot of UV-vis spectrum.


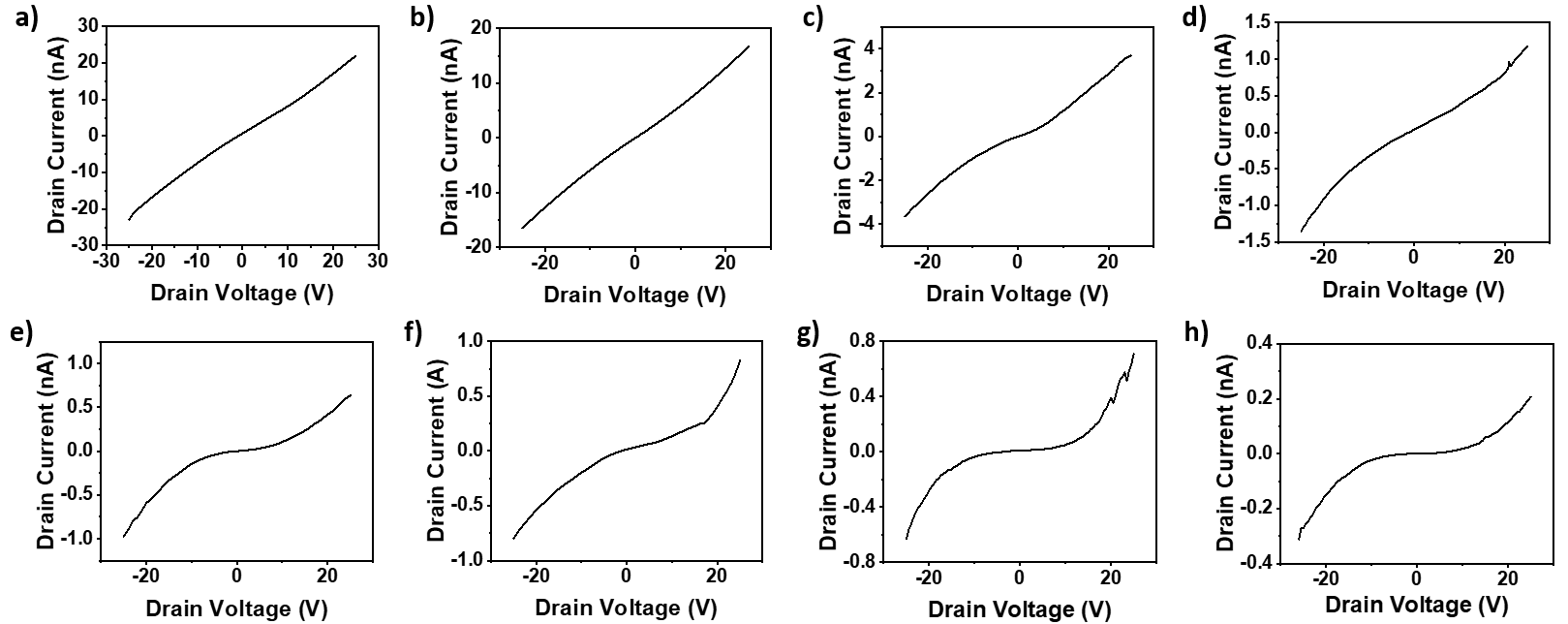


**Figure S9.** IV characteristic curve of the TAPB-PDA COF film at different channel lengths. (a) 9 μm, (b) 12 μm, (c) 15 μm, (d) 18 μm, (e) 21 μm, (f) 24 μm, (g) 27 μm, and (h) 30 μm.

**References**

[1] Perdew, J. P.; Zunger, A. J. P. R. B., *Phys. Rev. B* **1981,** *23* (10), 5048.

[2] Grimme, S.; Antony, J.; Ehrlich, S.; Krieg, H. J. T. J. o. c. p., *J. Chem. Phys.* **2010,** *132* (15).

[3] Kresse, G.; Hafner, J. J. P. r. B., *Phys. Rev. B* **1993,** *47* (1), 558.

[4] Kresse, G.; Furthmüller, J. J. P. r. B., *Phys. Rev. B* **1996,** *54* (16), 11169.

[5] Kresse, G.; Joubert, D. J. P. r. b., *Phys. Rev. B* **1999,** *59* (3), 1758.

[6] Kim, Y. Y.; Ree, B. J.; Kido, M.; Ko, Y. G.; Ishige, R.; Hirai, T.; Wi, D.; Kim, J.; Kim, W. J.; Takahara, A. J. A. E. M., *Adv. Electron. Mater.* **2015,** *1* (10), 1500197.

[7] Jiang, Z. J. J. o. A. C., *J. Appl. Crystallogr.* **2015,** *48* (3), 917-926.
